# Outcomes of Iso-Elastic Acetabular Cup in Primary Total Hip Arthroplasty with 5-Year Minimum Follow-Up: A Systematic Review

**DOI:** 10.3390/jcm14186621

**Published:** 2025-09-19

**Authors:** Vincenzo Longobardi, Marco Minelli, Giacomo Pietrogrande, Giuseppe Anzillotti, Federico Della Rocca, Mattia Loppini

**Affiliations:** 1 Department of Biomedical Sciences, Humanitas University, Via Rita Levi Montalcini 4, Pieve Emanuele, 20090 Milan, Italy; drv.longobardi@gmail.com (V.L.); giacomo.pietrogrande@humanitas.it (G.P.); giuseppe.anzillotti@humanitas.it (G.A.); mattia.loppini@hunimed.eu (M.L.); 2 IRCCS Humanitas Research Hospital, Via Manzoni 56, Rozzano, 20089 Milan, Italy; federico.della_rocca@humanitas.it

**Keywords:** isoelastic, cup, hip, arthroplasty

## Abstract

**Background:** Long-term survivorship in total hip arthroplasty (THA) is influenced by implant stability and stress distribution to surrounding bone. Isoelastic acetabular components are monoblock polyethylene cups with a low elastic modulus, which were developed to reduce stress shielding and enhance periacetabular bone preservation. This systematic review aimed to evaluate the mid- to long-term clinical outcomes, wear rate, and survivorship of isoelastic cups in primary THA with a minimum follow-up of five years. **Materials and methods:** A systematic literature search was performed in April 2025 across PubMed, Embase, Cochrane Library, and Google Scholar following PRISMA 2020 guidelines. Inclusion criteria comprised clinical studies on isoelastic acetabular cups in primary THA with a minimum of five years of follow-up. Data on survivorship, complications, clinical outcomes, wear, and radiological performance were extracted and analyzed. Risk of bias in each study was assessed through the Newcastle–Ottawa Scale (NOS) for observational studies and the Cochrane Risk of Bias 2 (RoB 2) tool for randomized controlled trials. **Results:** Twelve studies, encompassing 1491 hips, met the inclusion criteria. Mean follow-up was 8.1 years. Overall implant survival rate ranged from 82.7% to 100%. Mean Harris Hip Score was 92.6, with low reported pain and high satisfaction. Mean annual wear was 0.05 mm/year. Vitamin E-infused highly cross-linked polyethylene (VEHXLPE) cups demonstrated lower femoral head penetration compared to UHMWPE. A randomized trial showed reduced bone loss in the polar region with isoelastic cups versus modular titanium cups (4.9% versus 15.9%, *p* = 0.005). Complication and revision rates were low, though heterogeneity in cup positioning reporting and variable follow-up durations were noted. **Conclusions:** Isoelastic acetabular components demonstrate excellent survivorship, low wear rates, and favorable clinical outcomes at mid- to long-term follow-up. High-quality, long-term comparative studies are needed to confirm these findings across broader patient populations.

## 1. Introduction

Achieving long-term implant survival remains one of the most significant challenges in total hip arthroplasty (THA) [1]. Critical factors influencing implant longevity include immediate primary stability and surface properties that promote secondary stability through bony ingrowth [1]. In this context, uncemented acetabular components were introduced with the aim of enhancing biological fixation and improving long-term outcomes [2]. To enhance primary stability, cup designs with a larger equatorial diameter and a flattened pole have been adopted. These allow for press-fit fixation at the equator but often result in non-physiological load distribution across the acetabulum [3]. Evidence suggests that following THA, the surrounding bone is subjected to altered load transfer, which leads to bone remodeling and a significant reduction in bone mineral density (BMD), particularly medially and inferiorly to the acetabular component [4]. This acetabular bone density loss is referred to as retroacetabular stress shielding and could influence the long-term survival of the implants [5,6]. Notably, stiff cementless titanium cups have been associated with substantial periacetabular bone loss [7]. Retroacetabular stress shielding may be attenuated through the use of implants with an elastic modulus more closely matched to that of native bone, thereby promoting more physiological load transfer and potentially enhancing implant survivorship. This design principle has led to the development of isoelastic acetabular cups, which are monoblock polyethylene acetabular components coated with titanium particles, designed to approximate the elastic properties of cancellous bone [8]. Given recent advancements in biomaterials and the renewed interest in elastic design philosophies in THA, a critical evaluation of the clinical reliability and survivorship of isoelastic acetabular cups is warranted. The primary objective of this systematic review is to analyze isoelastic cups survival and complication rates at minimum five-year follow-up, with a secondary focus on assessing wear rates and clinical and implant-related mechanical outcomes.

## 2. Materials and Methods

**Figure 1:**
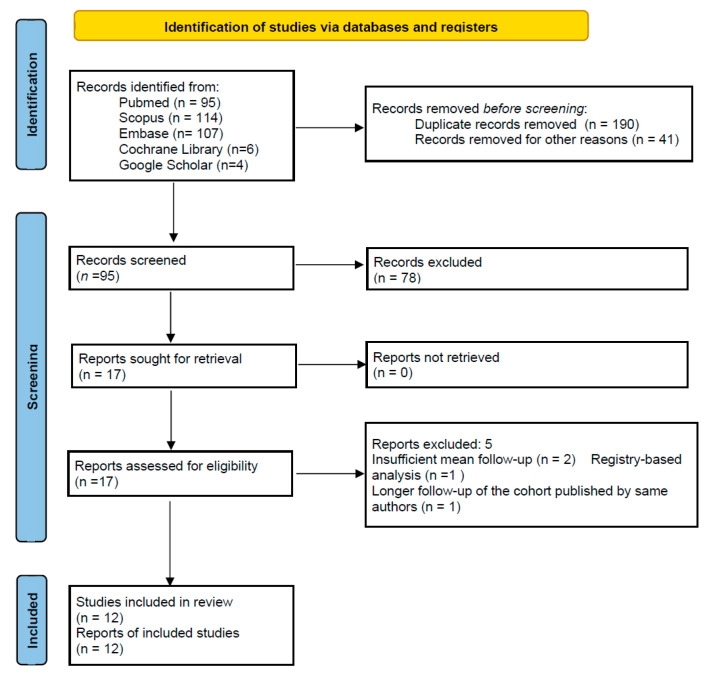
Flowchart of the selection process according to the PRISMA 2020 (Preferred Reporting Items for Systematic Reviews and Meta-Analyses) guidelines.

This systematic review was conducted in accordance with the Preferred Reporting Items for Systematic Reviews and Meta-Analyses (PRISMA) 2020 guidelines [9]. On 22 April 2025, a comprehensive literature search was performed across PubMed, Embase, Cochrane Library, and Google Scholar. The search strategy employed the following keywords and Medical Subject Headings (MeSH): (isoelastic OR “iso-elastic”) AND (cup OR hip). The search encompassed studies published from database inception to the date of the search and placed no restriction on language at the search level. The full electronic search strategy for PubMed is detailed in Appendix A, in accordance with PRISMA 2020 guidelines. The search strategy was peer-reviewed using the Peer Review of Electronic Search Strategies (PRESS) checklist. Two impartial observers, V.L. and M.M., carried out the screening and analysis independently. Any disagreements between reviewers were resolved through discussion and consensus. The articles were first filtered based on their title and abstract. The following inclusion criteria were applied during the screening process: (1) clinical studies; (2) studies written in English; (3) studies published in indexed journals; (4) full-text available; (5) studies in vivo; (6) studies on human subjects; (7) studies on isoelastic acetabular cups, defined as cups made from materials with a low elastic modulus approximating cancellous bone; (8) studies on primary total hip replacement; and (9) minimum follow-up of five years. Exclusion criteria encompassed (1) case reports, review articles, expert opinions, biomechanical studies, book chapters, and conference abstracts; (2) papers produced in languages other than English; (3) studies published in non-indexed journals; (4) studies in vitro; (5) trials conducted on animals; (6) studies on revision hip arthroplasty; and (8) minimum follow-up less than five years. The selected articles’ full texts were inspected in a second stage, and additional exclusions were made based on the previously mentioned criteria. All of the collected publications’ reference lists were further examined to find any possibly pertinent research. The process is outlined in a PRISMA flowchart (Figure 1). Data were extracted independently by the same two reviewers using a standardized data collection form. No automation tools were used. Extracted data included pertinent information on survivorship, revision and implant removal rates, complications (infection, dislocation, periprosthetic fracture, heterotopic ossification, loosening), clinical outcomes (Harris Hip Score, VAS score, Postel Merle d’Aubigné score, Oxford score), and implant-related mechanical performance (wear rates, femoral head penetration rate, bone mineral density). Data discrepancies were resolved by re-checking the original articles and consensus discussion between reviewers. All collected data were compiled into a dedicated database for synthesis and analysis. The Grading of Recommendations Assessment, Development, and Evaluation (GRADE) assessment was used to evaluate the strength of the recommendations and the quality of the evidence for the chosen outcomes. Based on risk of bias, inconsistency, indirectness, imprecision, and publication bias, every outcome was graded as at high, moderate, low, and very low risk of bias. Risk of bias was assessed independently by the same two reviewers. For observational studies, the Newcastle–Ottawa Scale (NOS) was used to evaluate selection, comparability, and outcome domains (Table 1) [9]. Randomized trials, if included, were assessed using the Cochrane Risk of Bias 2 (RoB 2) tool (Table 2) [9].

## 3. Results

The initial search identified 95 potentially relevant articles after duplicates were removed. Following title and abstract screening, 78 studies were excluded. Five additional articles were excluded after full-text assessment: in three cases due to insufficient mean follow-up, one study was excluded because it was a registry-based analysis and the fifth [22] because the same authors published a longer follow-up on the same cohort of patients which was included in the studies selected for qualitative synthesis [10]. Finally, 12 studies were analyzed [10,11,12,13,14,15,16,17,18,19,20,21]. Publication year ranged from 2008 to 2025. Two studies were level of evidence I, 8 studies were level of evidence II, and 2 studies were level of evidence IV. Risk of bias in each study was assessed through the Newcastle–Ottawa Scale for observational studies and using the Cochrane Risk of Bias 2 (RoB 2) tool for randomized controlled trials, and results are reported in Table 1 and Table 2.

Patient populations ranged from 47 [12] to 675 [15] subjects per study, with mean follow-up durations from 5.0 [18,21] to 19.4 [17] years. The isoelastic cup investigated in all the included studies is the RM Mathys cup (Mathys Ltd., Bettlach, Switzerland) and its evolutions through the years: the RM Pressfit cup [16,17,18,19,20,21] (introduced to market in 2002) and the RM Pressfit Vytamis cup [10,11,12,13,14,15,20,21] (introduced to the market in 2009). The RM Pressfit cup is a pre-assembled, uncemented, hemispherical monoblock cup composed of titanium particle-coated ultra-high-molecular-weight polyethylene (UHMWPE). It achieves primary stability through an equatorial press-fit [23,24], while secondary stability is ensured by bony integration of the titanium coating. Further, up to four screws can be inserted into predefined holes in case of insufficient acetabular coverage or whenever sclerotic bone is present. To decrease wear rates, the UHMWPE of RM Pressfit cup polyethylene was changed to vitamin E-infused highly cross-linked polyethylene (VEHXLPE) in the RM Vitamys cup, but the design remained unchanged.

In the encompassed studies, 562 RM Pressfit Cup [16,17,18,19,20,21] and 929 RM Vytamis cup [10,11,12,13,14,15,20,21] were followed up at a mean time of 8.07 years (range 5–19.3 years). Mean loss of follow-up was 22.53% (range 0–52.6%). Mean age was 66 (range 52.2–75.4 years) and mean BMI was 26.9 (range 26.1–27.9). Surgical approach was reported in 10 out of 12 studies (for a total of 907 hips) [10,11,12,13,16,18,19,20,21]: posterolateral approach was performed for 228 hips [13,19,20,21] (25.1%), anterior for 93 [13,16] (10.25%), anterolateral for 389 [10,11,12,13,16,20,21] (42.9%), direct lateral for 192 [13,20,21] (21.1%), and trans-trochanteric for 5 [19,21] (0.5%). In one study [17] it was reported to have used an antero-lateral or posterior approach, but it was not specified in which proportion. Patients’ characteristics are summarized in Table 3. 

### 3.1. Survivorship and Complications

At minimum five-year follow-up, survival rate ranged from 82.7 to 100.0%. Considering revision for aseptic loosening as the endpoint, survival rate ranged from 94.4 to 100.0%. In the cohort studied by Haefeli et al. [13], two implants were revised (revision rate 2%): one cup was repositioned and fixed with two screws due to malpositioning in the postoperative radiographs, and the other one was revised due to psoas impingement. Mahmood et al. [15] reported 10 acetabular cup revisions (revision rate: 1.5%) because of three traumatic periprosthetic acetabular fractures, three prosthetic joint infections, one impingement, and two dislocations. Portet et al. reported seven cup revisions (revision rate: 3.4%) [19]: five cups were removed due to recurrent dislocations, one for post-traumatic loosening, and one because of periprosthetic infection. In a randomized controlled trial comparing the RM Pressfit cup and the RM Vitamys cup, Massier et al. [20] reported that in the vitamin E-blended HXLPE group, two patients (revision rate: 2%) underwent revision surgery (one because of infection and one for recurrent instability), and in the UHMWPE group, two patients (revision rate: 2%) underwent revision surgery (one for cup malpositioning and one for recurrent instability). Ihle et al. [17] reported 14 acetabular cup revisions (revision rate: 15%) after a mean time of 12.3 years: seven cases were due to osteolysis, five cases of aseptic loosening, and two periprosthetic infections. A summary of all the complications is presented in Table 4.

### 3.2. Clinical Outcomes

Clinical results were investigated in different ways: 11 out of 12 studies [10,11,12,14,15,16,17,18,19,20,21] reported the Harris Hip Score (HHS) [25], in 6 cases [10,12,13,14,15,20] the Visual Analog Scale/Numeric Rating Scale (VAS/NRS) score was analyzed, 3 studies [16,17,21] calculated the Postel Merle d’Aubigné (PMA/MAP) score [26,27], in 1 case [13] the modified Harris Hip Score (mHHS) was assessed, in 1 cohort [19] the Oxford Score [28] was used, and 1 study [16] also reported Charnley class, Devane’s level of activity, postoperative hip disability, and osteoarthritis outcome score (HOOS), WOMAC score, and SF12 life quality. Mean preoperative HHS was 53.2 (range: 33.7–61.1), mean preoperative rest pain VAS was 4.3 (range: 3.3–5), mean preoperative load pain VAS was 7.2 (range: 6.6–7.7), and mean VAS satisfaction was 2.6 (range: 1.7–3.8). Mean postoperative HHS was 92.7 (range: 84.7–98), mean postoperative rest pain VAS was 0.3 (range 0–0.7), mean postoperative load pain VAS was 0.8 (range 0.2–1.3), and mean satisfaction rate was 9.2 (range 8.6–9.9). A significant improvement compared to preoperative values was reported in all the other clinical scores. Clinical outcomes are shown in Table 5.

### 3.3. Implant-Related Outcomes

Concerning the implant-related outcomes over time, different methods were employed. In one study [12], included patients underwent a dual X-ray absorptiometry (DEXA) exam to assess periacetabular bone mineral density (BMD). Cup migration and polyethylene wear were assessed in three studies [10,11,18] on radiographs using the Einzel-Bild-Röntgen-Analyse (EBRA) method [29]. Femoral head penetration (FHP) rate, a parameter used to assess and predict wear rates and osteolysis, was evaluated with radiostereometric analysis (RSA) [30] conducted on radiographs made with the patient in the standing position in the study by Rochcongar et al. [21]; in other cases, FHP rate and mean wear rates were calculated manually on radiographs. Six out of twelve [11,15,16,19,20,21] studies reported the orientation of the implanted acetabular cup: mean values are reported in Table 3. Three studies [14,20,21] evaluated femoral head penetration (FHP) on radiographs using different methods: one study with the modified dual-circle technique previously described by Geerdink et al. [31,32], another study [20] with the MHP software, and Rochcongar et al. [21] performed it with RSA. Three clinical trials [10,11,18] using the EBRA method assessed a mean cup migration of 1.48 mm (range 1.24–1.7 mm) at a mean time of 7.1 years. At a mean follow-up of 5.5 years, the cumulative femoral head penetration was 0.29 (range 0.24–0.38 mm) mm for the RM Vitamys Cup (made of HXLPE) and 0.44 range (0.44–0.45 mm) for the RM PressFit Cup (made of UHMWPE). Mean wear was measured in six studies (in three cases [10,11,18] using EBRA method, Erivan et al. [16] assessed it with MHP software as described by Sarry [33,34], Rochcongar et al. [21] used the RSA, and in one case [19] the method of Livermore et al. [35] was used): at a mean follow-up of 7.3 years, the mean annual wear rate was 0.05 per year (range 0.02–0.09 mm/year). Implant-related outcomes are shown in Table 5.

### 3.4. Quality Assessment

A total of six outcomes among all the studies were assessed by GRADE (Table 6). Revision for aseptic loosening and complications rates maintained high quality of evidence, while implant survivorship, annual wear rate, and functional outcomes (HHS) were downgraded to moderate quality of evidence. Finally, periacetabular BMD preservation presented low quality.

## 4. Discussion

This systematic review aimed to evaluate the clinical outcomes, implant-related performance, and survivorship of isoelastic acetabular components in primary total hip arthroplasty (THA), with a minimum follow-up of five years. Our findings demonstrate that isoelastic cups, specifically the RM family (RM Pressfit and RM Vitamys), provide excellent mid- to long-term survivorship, with overall survival rates ranging from 82.7 to 100% and aseptic loosening-free survival rates ranging from 94.4 to 100.0%. Clinical outcomes were consistently favorable, and mean annual wear rates were low across studies, reinforcing the reliability of these implants.

The concept of isoelasticity in acetabular cup design stems from the goal of reproducing physiological load transmission patterns within the pelvis by closely matching the elastic modulus of the implant to that of the surrounding cancellous bone. Elastic modulus (also known as Young’s modulus) is a critical property that defines the stiffness of a material, which is its resistance to elastic deformation under load. Native cancellous bone in the pelvis typically exhibits an elastic modulus ranging between 0.1 and 2 GPa, depending on location, density, and patient-specific factors [36,37]. In contrast, traditional uncemented titanium alloy acetabular components possess an elastic modulus of approximately 110 GPa [38]. This mismatch between the implant and surrounding bone results in non-physiological load distribution, often concentrating stresses at the cup periphery while unloading the medial wall of the acetabulum. This phenomenon, known as retroacetabular stress shielding, has been well-documented radiographically and histologically, and it contributes to localized bone resorption, osteolysis, and potential implant loosening over time [39]. The RM Pressfit and RM Vitamys are manufactured from ultra-high-molecular-weight polyethylene (UHMWPE) or vitamin E-blended highly cross-linked polyethylene (VEHXLPE), materials with an elastic modulus more closely aligned with cancellous bone, reported around 0.5 to 1 GPa. Biomechanical studies have confirmed that materials with lower stiffness transfer more load to the periacetabular bone, particularly the medial wall, and thereby reduce stress shielding effects [40]. These findings align with earlier reports that stiff titanium-backed cups can lead to up to 33% BMD reduction relative to baseline [5]. One clinical study included in this review [12] utilized dual-energy X-ray absorptiometry (DEXA) to quantitatively assess periacetabular bone mineral density (BMD) in patients implanted with isoelastic cups, suggesting a more physiological pattern of periacetabular bone remodeling, with relative preservation of BMD in regions commonly affected by stress shielding. Moreover, the monoblock design of these cups eliminates the modular metal backing found in many contemporary components, thereby removing a potential site of micromotion and backside wear [41]. The absence of modularity may further contribute to a more homogeneous implant–bone interaction during dynamic loading, reducing the risk of fibrous interface formation and long-term loosening [42]. Additionally, the absence of dome holes may reduce the risk of acetabular osteolysis by limiting the migration of polyethylene wear debris to the subchondral bone [43]. Brodt et al. [3] found in a RCT that while overall periacetabular BMD loss was similar between groups, the isoelastic RM cup showed significantly less bone loss in the polar region (4.9% versus 15.9%, *p* = 0.005) compared to a modular titanium cup. However, monoblock polyethylene isoelastic cup positioning can be challenging: these implants lack polar screw holes, thus preventing direct visualization of proper seating [43]. Moreover, once impacted, the monoblock design does not allow intra-operative adjustment if the initial positioning is suboptimal. Additionally, the polyethylene component can deform under impaction, further complicating intraoperative positioning [44].

This review confirms the clinical viability of isoelastic acetabular cups, particularly when highly cross-linked polyethylene (HXLPE) infused with vitamin E is utilized. However, this could potentially introduce a bias in the present study, since the RM Pressfit Cup and the RM Vytamis Cup are made of different material, with the latter being the evolution of the first one. Indeed, the HXLPE of the RM Vytamis Cup is made of the same RM Pressfit Cup UHMWPE-Powder, but this is blended with 0.1% α-tocopherol (vitamin E) before it is consolidated and cross-linked, resulting in a homogenous distribution [45]. The addition of vitamin E protects the HXLPE against oxidation, leading to lower wear rates of vitamin E irradiated polyethylene (VEPE) cups as compared to UHMWPE cups [46]. These data are confirmed by a RCT of Massier et al. [20], who reported that the mean FHP rate of the vitamin E blended HXLPE cup after 6 years was lower compared with the FHP rate of the UHMWPE cup (*p* = 0.002). Further, a meta-analysis by Cheng et al. [47] showed that the addition of vitamin E to highly cross-linked polyethylene liners in THA could reduce the all-cause revision rate by approximately 46% in the short-term follow-up.

Overall complication and revision rates were observed to be low. The most common reasons for cup revision were dislocation, infection, malposition, and impingement. Aseptic loosening and osteolysis were uncommon, further supporting the mechanical integrity and osteointegrative capacity of these implants. Notably, one outlier study [17] reported a higher revision rate (15%), primarily due to osteolysis and aseptic loosening; however, this may reflect longer follow-up (mean 12.3 years) and underscores the importance of continued long-term surveillance. In particular, aseptic loosening is a multifactorial complication in total hip arthroplasty, often resulting from a combination of biological and mechanical factors: contributing elements include polyethylene wear and particle-induced osteolysis [48,49,50,51], as well as insufficient cup press-fit [52], malpositioning [53], and stress shielding [54]. These factors collectively make aseptic loosening the leading cause of revision THA [54]. Importantly, they also highlight a limitation of the present review: only 6 out of 12 studies reported acetabular cup positioning. Of these, two studies [11,15] stated that components were placed within the Lewinnek “safe zone” [53], but did not provide specific angle measurements, while three others reported only inclination, not mentioning anteversion. This variability in reporting may affect the reproducibility and generalizability of the overall findings. Only three studies [10,17,19] reported a follow-up longer than 10 years, with survival rate ranging from 96.1% to 100%. These findings are similar to data from national joint replacement registries [55] and in line with other implants, such as hemispheric titanium porous-coated acetabular components [56].

Clinical outcomes were consistently positive, with low postoperative pain scores (mean VAS rest pain: 0.3; load pain: 0.8), high satisfaction rates, and mean postoperative Harris Hip Scores exceeding 90 points in most studies (overall mean HHS was 92.6). These findings indicate durable functional improvement and pain relief, corroborating the mechanical and biological stability inferred from radiographic and wear data.

Nevertheless, the current evidence base is subject to several limitations. First, all included studies assessed a single implant family (RM series), potentially limiting the generalizability of the findings to other isoelastic designs. Second, considerable heterogeneity exists in study methodology, patient demographics, follow-up duration, and outcome reporting. Wear analysis techniques varied widely, including manual radiographic assessment, EBRA, and RSA, which complicates direct comparison across studies. Additionally, loss to follow-up was moderate (mean 22.5%), introducing the possibility of attrition bias. While several studies were of high methodological quality (including two level I trials), the majority were level II, with some level IV evidence included.

Despite these limitations, the strengths of this review lie in its rigorous methodological framework, adherence to PRISMA guidelines, and comprehensive synthesis of long-term data. To our knowledge, this is the first systematic review dedicated exclusively to isoelastic acetabular components with a minimum five-year follow-up, providing valuable insights into their mid- and long-term performance.

Looking ahead, future research should aim to address the current gaps by conducting high-quality randomized controlled trials directly comparing isoelastic cups to conventional titanium-backed modular designs, particularly with respect to stress shielding, implant migration, and bone mineral density. Standardization of wear assessment methodologies, broader reporting of pre- and postoperative functional outcomes, and extended follow-up beyond 20 years will be critical to validating the longevity of isoelastic designs. Additionally, evaluating outcomes in younger, more active patient populations should be prioritized, since these patients may particularly benefit from bone-preserving implants.

In conclusion, isoelastic acetabular components demonstrate excellent survivorship, low complication rates, and satisfactory functional outcomes in mid- to long-term follow-up. Their favorable biomechanical profile, including low wear and potential for improved load transfer, makes them a compelling option in contemporary total hip arthroplasty. Continued clinical monitoring and future prospective studies are essential to confirm these findings over longer durations and across broader patient populations, particularly those at greater risk of bone loss or requiring long-term implant durability.

## Figures and Tables

**Table 1:** Newcastle–Ottawa quality assessment scale for cohort studies.

Study	Selection: Representativeness of Exposed Cohort (★)	Selection: Selection of Non-Exposed Cohort (★)	Selection: Ascertainment of Exposure (★)	Selection: Outcome Not Present at Start (★)	Comparability: Control for Confounding (★★)	Outcome: Assessment of Outcome (★)	Outcome: Follow-Up Long Enough (★)	Outcome: Adequacy of Follow-Up (★)	Total Stars	Notes
**Afghanyar et al., 2024** [10]	**★**	**×**	**★**	**★**	**×**	**★**	**★**	**★**	6	Prospective design; no control group; validated and standardized outcome measures; 10-year follow-up; high % of loss to follow-up, 29.7%, but good description provided of those lost
**Afghanyar et al., 2023** [11]	**★**	**★**	**★**	**★**	**★**	**★**	**★**	**★**	8	Retrospective matched-pair study; time lapse during which patients underwent surgery was not reported; groups matched by sex, age, body mass index (BMI), and ASA classification; EBRA method on radiographs; mean follow-up: 5 years
**Anderl et al., 2022** [12]	**★**	**×**	**★**	**★**	**×**	**★**	**★**	**★**	6	Prospective design; no control group; small cohort: 47 patients; 5-year follow-up with clinical and radiographic outcomes; lack of adjustment for potential confounder; dual-energy X-ray absorptiometry used to assess instrumental outcomes
**Haefeli et al., 2025** [13]	**★**	**×**	**★**	**★**	**×**	**★**	**★**	**★**	6	Prospective design; no control group; use of validated tools to assess outcomes; 10-year follow-up; 38% loss of follow-up (though well documented) and cross-checked with national registry (SIRIS) improves ascertainment of revisions
**Snijders et al., 2020** [14]	**★**	**×**	**★**	**★**	**×**	**★**	**★**	**★**	6	Prospective design; clear inclusion criteria; no control group; surgical approach not reported; standardized clinical and instrumental outcomes assessment; 6-year mean follow-up; loss of follow-up was 15.2%, but good description provided of those lost
**Mahmood et al., 2014** [15]	**★**	**×**	**★**	**★**	**×**	**★**	**★**	**★**	6	Prospective multicenter study; standardized data collection; validated clinical outcomes and radiographic monitoring; no control arm; no adjustment for potential confounders; long mean follow-up (8.9 years) with detailed survival analysis; 66.7% of enrolled patients completed clinical/radiographic follow-up
**Erivan et al., 2020** [16]	**★**	**×**	**★**	**★**	**×**	**★**	**★**	**★**	6	Prospective design; no control group; validated clinical score to assess outcome; radiographic evaluation was not blinded; mean follow-up: 6.5 years with low rate of loss of follow-up (2.1%); use of survival analysis with clear endopoints; no statistical adjustment for confounders
**Ihle et al., 2008** [17]	**★**	**×**	**★**	**★**	**×**	**★**	**★**	**★**	6	Prospective series with clinical and radiographic evaluation; no comparator group; subgroup analyses by head size and age groups were performed; long mean follow-up: 19.3 years; high percentage of loss of follow-up (52.6%), but good description provided of those lost
**Wyss et al., 2013** [18]	**★**	**×**	**★**	**★**	**×**	**★**	**★**	**★**	6	Prospective multicenter study; small cohort; 5-year radiographic and clinical analysis; EBRA method used on radiographs; no comparator and 28% loss of follow-up (even if good descriptions were provided)
**Portet et al., 2024** [19]	**★**	**×**	**★**	**★**	**×**	**★**	**★**	**★**	6	Retrospective study; validated tools to assess clinical and radiographic outcomes; 10.5-year data; limited by absence of control group and absence of adjustment for potential confounders; radiological measurements were performed by a single observer.

★ = Criterion fulfilled (1 point), ★★ = Criterion exceptionally fulfilled (2 points; where applicable), × = Criterion not fulfilled or insufficiently reported

**Table 2:** Cochrane Risk of Bias 2 (RoB 2) tool for randomized control trial.

Study	Randomization Process	Deviations from Intended Interventions	Missing Outcome Data	Measurement of the Outcome	Selection of the Reported Result	Overall Risk of Bias	Notes
**Massier et al., 2020** [20]	Low risk	Some concerns	Some risk	Low risk	Low risk	Low risk	Well-randomized; allocations performed using an internet randomization system; standardized procedures; blinded assessors; surgeons were not blinded; high rate of loss of follow-up: 11%; the HXLPE group received significantly larger head sizes due to thinner polyethylene liners
**Rochcongar et al., 2021** [21]	Low risk	Some concerns	Low risk	Low risk	Low risk	Some risk	Validated RSA method to assess wear rate; small sample size with further loss of follow-up (10 patients): only 40 patients followed up at 5 years; high-quality design despite unblinded surgeons; surgical approach was not reported

**Table 3:** Patients’ characteristics and demographics in the included studies.

Author	Year	Patients	Total Hips	Cup	FU Hips	Lost to FU (%)	Females	Males	Age (Years)	BMI
**Afghanyar et al.** [10]	2024	96	101	RM Pressfit vitamys cup (HXLPE)	71 (57 both clinical and radiological FU + 14 only clinical FU)	29.7% (7 lost, 23 deaths not related to primary THA)	63	38	69.4 (50.7–84.3)	27.5 (19.3–41.5)
**Afghanyar et al.** [11]	2023	98	98	RM Pressfit vitamys cup (HXLPE)	98	0	52	46	67.1 (50.7–78.7)	26.8 (4.03)
**Anderl et al.** [12]	2022	47	47	RM Pressfit vitamys cup (HXLPE)	41	12.7% (3 lost, 3 deaths not related to primary THA)	23	22	66.8 (46.0–80.0)	/
**Hefaeli et al.** [13]	2025	150	162	RM Pressfit vitamys cup (HXLPE)	100 (99 both clinical and radiological evaluation, 1 only radiological)	38.3% (20 deaths not related to primary THA, 42 lost)	74	76	67.2 (38–88)	27.3 (16.7–46.9)
**Snijders et al.** [14]	2019	112	117	RM Pressfit Vitamys cup (HXLPE)	100	15.2% (4 deaths not related to primary THA, 13 lost)	74	38	63.8 (40.0–86.0)	26.15 (19.0–35.0)
**Massier et al.** [20]	2020	199 (102 vit E blended HXLPE cup, 99 UHMWPE cup)	199	RM PressFit Vitamys cup (HXLPE) and RM Pressfit cup (UHMWPE)	177	11%	141	36	65	/
**Mahmood F. F. et al.** [15]	2021	675	675	RM Pressfit vitamys cup (HXLPE)	450	33.3% (53 deaths not related to primary THA, 172 lost)	395	280	68.3 (34.7–93.1)	27.5 (14.6–46.9)
**Erivan et al.** [16]	2016	189	189	RM Pressfit cup (UHMWPE)	185 of which only 101 with X-rays	2.1% (4 lost)	108	81	75.4 (29.0–90)	26.1 (15–39)
**Ihle et al.** [17]	2008	93	93	RM Pressfit cup (UHMWPE)	44	52.6% (25 deaths not related to primary THA, 14 revised, 5 lost)	41	39	52.2 (28.0–81)	26.3 (18.7–36.3)
**Wyss et al.** [18]	2013	50	50	RM Pressfit cup (UHMWPE)	36	28% (8 deaths not related to primary THA, 6 lost for other reasons)	22	28	72.3 (54.1–90.1)	27.9 (16.3–39.2)
**Rochcongar et al.** [21]	2021	62	33 HXLPE; 29 UHMWPE	RM Pressfit vitamys cup (HXLPE) or RM Pressfit cup (UHMWPE)	22 HXLPE; 18 UHMWPE	33% HXLPE group; 37,9% UHMWPE	16 HXLPE group; 17 UHMWPE group	16 HXLPE group; 12 UHMWPE group	61	27
**Portet et al.** [19]	2024	163	207	RM Pressfit cup (UHMWPE)	182 hips included for survival analysis, 157 hips included for clinical and radiological evaluation	12%	60	103	63	27 (24.5–30)

**Table 4:** Minimum five-year follow-up outcomes: complications.

Authors	Followed-Up Hips	Infections	Hematoma	Nerve Injury/Palsy	Periprosthetic Fractures	Heterotopic Ossifications	Aseptic loosening	Osteolysis	Dislocation
**Afghanyar et al. (2024)** [10]	71	1 superficial	4	1	2	6	0	0	/
**Afghanyar et al. (2023)** [11]	49	/	/	/	/	1 (VEPE)	0	0	/
**Anderl et al.** [12]	41	0	0	0	0	0	0	0	1
**Hefaeli et al.** [13]	100	0	0	0	0	35	0	0	/
**Snijders et al.** [14]	100	3 superficial, 2 deep	0	0	1 femoral	0	0	0	/
**Massier et al.** [20]	199	2 superficial, 1 deep	0	2	2 femoral, 1 acetabular	/	0	0	6
**Mahmood et al.** [15]	450	3 deep	0	10	12	/	0	0	8
**Erivan et al.** [16]	189	4	1	1	0	1	0	0	2
**Ihle et al.** [17]	93	1 superficial, 1 deep	0	0	18 femoral	25	5	8	2
**Wyss et al.** [18]	36	/	/	/	1	10	0	1	/
**Rochcongar et al.** [21]	40	0	0	0	0	0	0	0	0
**Portet et al.** [19]	182	1 deep	0	0	0	0	1 post-traumatic	0	5

**Table 5:** Minimum five-year follow-up outcomes: clinical and implant-related outcomes.

Authors	Cup	Surgical Approach	Score	Mean Follow-Up	Clinical Results	Cup Positioning	Implant-Related Outcomes	FHP Rate	Mean Cup Migration	Mean Wear	Survivorship
**Afghanyar et al. (2024)** [10]	RM Pressfit vitamys cup (HXLPE)	Antero-lateral approach	HHS, VAS	129.3 months (120.0 to 148.9)	Mean HHS improved from 49.9 to 96.4 (*p* = 0.052). Mean rest pain VAS decreased from 5.0 to 0.0. Mean load pain VAS decreased from 7.7 to 0.2. Mean satisfaction increased from 1.7 to 9.9.	/	Mean cup migration at 5 years was 1.34 mm; it increased to 1.67 mm at 10 years. During the critical first year, we observed a mean cup migration of 0.80 mm.	/	The annual cup migration rate decreased from 0.30 mm at 5 years to 0.16 mm at 10 years.	Mean total wear at last follow-up was 0.35 mm; the measured values decreased slightly from the five-year results at 0.40 mm. Mean annual wear rate was 0.03 mm per year.	100% (Lost to FU patients not excluded)
**Afghanyar et al. (2023)** [11]	RM Pressfit vitamys cup (HXLPE)	Antero-lateral approach	HHS	73.2 ± 19.2 months	Mean HHS improved significantly.	Within the Lewinnek safe zone: cup inclination of 40 ± 10° and anteversion of 15 ± 10°	Mean cup migration at the mid-term follow-up was 67 ± 0.92 mm while the annual migration rate was 0.27 ± 0.16 mm/year at 5 years.	/	Mean cup migration at the mid-term follow-up was 1.67 ± 0.92 mm while the annual migration rate was 0.27 ± 0.16 mm/year at 5 years.	Mean total wear at the mid-term follow-up was 0.37 ± 0.28 mm and the mean annual wear rate was 0.06 ± 0.04 mm/year.	100% (Lost to FU patients not excluded)
**Anderl et al.** [12]	RM Pressfit vitamys cup (HXLPE)	Antero-lateral approach	HHS, VAS	63.7 months (12.2–68.1)	Mean HHS improved significantly from 56.5 to 95.7 (*p* < 0.0001). Mean rest pain VAS decreased from 4.5 to 0.7. Mean load pain VAS decreased from 7.2 to 1.3 and satisfaction increased from 1.9 to 9.6.	/	On the acetabular side, DEXA evaluation revealed BMD stabilized in all DeLee and Charnley zones after an initial postoperative decrease. There were non-significant differences in the BMD in DeLee and Charnley zones II and III between 24 and 60 months after surgery (*p* > 0.05). In zone I, the BMD significantly differed between 24 and 60 months after surgery, with an estimated decrease of 4%.	/	/	/	100% (Lost to FU patients excluded)
**Hefaeli et al.** [13]	RM Pressfit vitamys cup (HXLPE)	85.8% anterior, 9.9% anterolateral, 3.7% transgluteal, 0.6% posterior	mHHS, VAS	120.5 months (118.0–126.0)	Mean rest pain VAS decreased from 3.3 to 0.1. Mean load pain VAS decreased from 6.6 to 0.5 and satisfaction increased from 3.8 to 9.5. Mean mHHS was 94.8, with a mean improvement compared to preoperative mHHS of 33.7.	/	At the ten-year radiographic follow-up, no signs of loosening, acetabular radiolucent lines or osteolysis were observed.	/	/	/	Survival rate was 98%. Survival rate for aseptic loosening was 100%. (Lost to FU patients not excluded)
**Snijders et al.** [14]	RM Pressfit Vitamys cup (HXLPE)	/	HHS, VAS	71.4 months (57.8–82.4)	Mean rest pain VAS decreased from 4.53 a to 0.45. Mean load pain VAS decreased from 7.41 to 1.26. Mean satisfaction increased from 3.09 to 8.75. Mean HHS increased from 61.1 to 91.8.	/	Mean total FHP on radiographs was 0.249 mm. Mean FHP rate was 0.036 mm/year.	Mean total FHP was 0.249 mm. Mean FHP rate was 0.036 mm/year.	/	/	Survival rate was 97.4%. Survival rate for aseptic loosening was 100%. (Lost to FU patients not excluded)
**Massier et al.** [20]	102 RM PressFit Vitamys cup (HXLPE) and 97 RM Pressfit cup (UHMWPE)	49% lateral, 43% posterolateral, 8% anterolateral	VAS, HHS	70.0 months	Mean preoperative NRS scores for rest pain, load pain, patient satisfaction were around 4, 6, and 4 (only graphically reported). Mean postoperative NRS scores for rest pain, load pain, and patient satisfaction were 0.3, 0.6, and 8.6, respectively. Mean HHS increased from 60 to 93.	Inclination: <35°: 8%; 35–40°: 24%; 41–45°: 27%; 46–50°: 26%; >50°: 16%	Mean FHP rate on radiographs of the HXLPE/VitE cup was lower compared with the FHP rate of the UHMWPE cup (*p* = 0.002). Multivariate analysis showed no statistically significant effect of head size on the wear rates in either cup.	Total mean FHP was 0.38 mm in the HXLPE/VitE cup and 0.44 mm in the UHMWPE cup (*p* = 0.01). Mean FHP rate was 0.028 and 0.035 mm/year for the HXLPE/VitE cup and UHMWPE cup, respectively.	/	/	Survival to revision was 98% for both groups. Survival rate for aseptic loosening was 100% in both groups. (Lost to FU patients not excluded)
**Mahmood F. F. et al.** [15]	RM Pressfit vitamys cup (HXLPE)	/	HHS, VAS	105.0 months	Mean HHS increased from 54.1 to 93.8. Mean rest pain VAS decreased from 4.5 to 0.3. Mean load pain VAS decreased from 7.2 to 0.7. Mean patient satisfaction improved from 2.7 to 8.9 at 6–12 weeks and was maintained through the 5-year follow-up.	89% of acetabular cups within the Lewinnek safe zone	A single case with a radiolucent line in zone 2 was identified. There was no evidence of acetabular osteolysis on radiographs in DeLee and Charnley zones 1–3 throughout the follow-up period.	/	/	/	Survival rate was 98.9%. Survival rate for aseptic loosening was 100%. (Lost to FU patients not excluded)
**Erivan et al.** [16]	RM Pressfit cup (UHMWPE)	96.3% anterolateral, 3.7% anterior	PMA, HHS, Charnley class, Devane’s level of activity, HOOS, WOMAC, SF12	6.5 years (5.0–8.0)	Mean PMA score increased 11.4 to 15.8. Mean HHS improved from 56.8 to 84.7 (*p* < 0.001). Devane classes were preoperatively distributed with 5 patients (4.9%) in class 1, 33 (32%) in class 2, 51 (49.5%) in class 3, 13 (12.6%) in class 4, and 1 (1%) in class 5. Postoperative scores were distributed with 13 (12.6%) in class 1, 24 (23.3%) in class 2, 49 (47.7%) in class 3, 15 (14.6%) in class 4, and 2 (1.9%) in class 5. Postoperative HOOS was measured in 109 patients with the mean at 75.9. WOMAC index was measured at late follow-up with a mean of 23.7. SF-12 quality of life score was found to be 38.3 for the Physical Composite Score (11.6–63.1) and 47.4 for the Mental Composite Score (12.9–72.9).	Mean cup anteversion was 16.3° (0–35.5°). Mean inclination was 42.3° (24–62°).	Mean annual wear rate was calculated on radiographs by MHP software (version not specified) and was found to be 0.065 mm per year.	/	/	Mean annual wear rate was calculated on radiographs by MHP software and was found to be 0.065 mm per year.	Survival rate was 96.8%. Survival rate for aseptic loosening was 100%. (Lost to FU patients not excluded)
**Ihle et al.** [17]	RM Pressfit cup (UHMWPE)	Anterolateral or posterior	HHS, PMA	19.3 years (17.4–20.9)	Mean HHS was 87.5 at the latest FU. Mean PMA pain score improved from 2.2 to 5.8, mobility from 4.5 to 5.8, ability to walk from 3 to 4.6.	/	A radiolucent line adjacent to the acetabular component was seen in DeLee and Charnley zone 1 on two radiographs, and in zone 3 on one radiograph. None had continuous radiolucencies in all three zones. Osteolysis was seen as a sharply demarcated radiolucent space on eight radiographs: six in zone 3, one in zone 2, and one in zone 1. None of these patients showed any clinical signs of loosening and none are awaiting revision.	/	/	/	Survival rate was 82.7%. Survival rate for aseptic loosening was 94.4%. (Lost to FU patients not excluded)
**Wyss et al.** [18]	RM Pressfit cup (UHMWPE)	Transgluteal	HHS	60 months	Preoperatively, 90% of the patients had a poor HHS and 10% a moderate HHS. Five years after surgery, 30 patients were examined: 77% reached an excellent, 7% a good, 3% a moderate, and 13% a poor HHS.	/	Mean cup migration rate and annual wear rate analysis revealed a decrease in speed of both phenomenon over time.	/	Mean cup migration was 1.25 mm	The mean annual wear rate was 0.09 mm/year.	100% (Lost to FU patients not excluded)
**Rochcongar et al.** [21]	RM Pressfit vitamys cup (HXLPE) or RM Pressfit cup (UHMWPE)	51.6% anterolateral, 43.5% posterior, 4.8% transtrochanteric	HHS, PMA	60 months	Mean HHS and the MAP score improved in both groups (*p* < 0.001). None of the mean clinical scores differed significantly between the HXLPE/VitE group and the UHMWPE group.	Mean cup inclination angle was similar in both groups (HXLPE/VitE 48° UHMWPE 46°) and remained stable over the entire follow-up period.	Mean femoral head penetration increased 0.08 mm in HXLPE/VitE cups, while it increased 0.2 mm in UHMWPE cups per year. The estimated steady-state rate of wear was approximately 66% lower in the HXLPE/VitE group than in the UHMWPE group (*p* < 0.001).	Cumulative FHP was 0.24 in the HXLPE/VitE group and 0.45 mm in the UHMWPE group	/	The wear rate averaged 0.02 mm/year in the HXLPE/VitE group compared with 0.06 mm/year in the UHMWPE group. In the HXLPE/VitE group, the wear rate was 66% lower than in the UHMWPE group. Both groups showed no significant correlation of PE wear with cup inclination angles.	100% (Lost to FU patients excluded)
**Portet et al.** [19]	RM Pressfit cup (UHMWPE)	69% posterolateral, 30% lateral, 1% transtrochanteric	HHS, Oxford score	10.5 years	Median postoperative HHS and Oxford scores were 95 (90–98) and 19 (17–23).	Mean inclination was 48° (45−52)	Radiographic analysis showed that 16% of cups exhibited wear greater than 1 mm. In the immediate postoperative period, 27% of cups were not press-fit at the level of the acetabular back rim, 4% of cups were not press-fit at the level of the acetabular roof, and 24% of cups were not press-fit at either location. At 10 years of follow-up, 6% of cups retained a gap at the back rim, and 3% at both locations. At 10 years of follow-up, 3.2% showed periprosthetic osteolysis, and 4.5% had geodes.	/	/	Polyethylene wear on radiographs was 0.058 mm/year. Twenty-five (16%) cups exhibited wear greater than 1 mm at 10 years.	Survival rate was 96.1%. Survival rate for aseptic loosening was 99.5%. (Lost to FU patients excluded)

**Table 6:** GRADE certainty of evidence table.

Outcome	No. of Studies	Study Design	Risk of Bias	Inconsistency	Indirectness	Imprecision	Publication Bias	Overall Certainty	Effect/Finding
**Implant Survivorship (≥5 years)**	12	Observational + 2 RCTs	Moderate	Low	No	Low	Not detected	⬤⬤⬤◯Moderate	Pooled survivorship 97.5% (99.5% excluding septic causes)
**Revision for Aseptic Loosening**	11	Observational + RCTs	Low	Low	No	Low	Not detected	⬤⬤⬤⬤High	<1% revision rate for aseptic loosening
**Annual Wear Rate (mm/year)**	6	Observational + RCTs	Moderate	Moderate	No	Moderate	Not detected	⬤⬤⬤◯Moderate	Mean: 0.05 mm/year (range 0.02–0.09); VEHXLPE < UHMWPE
**Functional Outcome (HHS)**	11	Observational + RCTs	Moderate	Moderate	No	Moderate	Not detected	⬤⬤⬤◯Moderate	Mean postop HHS: 92.6; significant improvement
**Complication Rates**	12	Observational + RCTs	Low	Low	No	Low	Not detected	⬤⬤⬤⬤High	Low rates: dislocation, infection, fracture
**Periacetabular BMD Preservation**	1	RCTs	Some concerns	High	No	High	Not detected	⬤⬤◯◯Low	1 RCT showed reduced BMD loss in polar region (*p* = 0.005)

⬤ = Point showing level of certainty, ◯ = Empty point (not awarded); Certainty of evidence is rated as: ⬤⬤⬤⬤ = High; ⬤⬤⬤◯ = Moderate; ⬤⬤◯◯ = Low.

## Data Availability

Not applicable.

## Appendix A

**Database:** PubMed

**Date of Search:** 22 April 2025

**Search Query:**

(isoelastic OR “iso-elastic”) AND (cup OR hip)

**Filters applied:**

- No language restrictions
- From inception to 22 April 2025

The same search strategy was adapted and used for Embase, Cochrane Library, and Google Scholar.
